# Reformulation of Muffins Using Inulin and Green Banana Flour: Physical, Sensory, Nutritional and Shelf-Life Properties

**DOI:** 10.3390/foods10081883

**Published:** 2021-08-15

**Authors:** Rania Harastani, Lewis J. James, Sourav Ghosh, Andrew J. Rosenthal, Elliot Woolley

**Affiliations:** 1 Wolfson School of Mechanical, Electrical and Manufacturing Engineering, Loughborough University, Loughborough LE11 3TU, UK; S.Ghosh2@lboro.ac.uk (S.G.); e.b.woolley@lboro.ac.uk (E.W.); 2 School of Sport and Exercise Science, Loughborough University, Loughborough LE11 3TU, UK; L.James@lboro.ac.uk; 3 School of Biosciences, University of Nottingham, Nottingham LE12 5RD, UK; DocARosenthal@gmail.com

**Keywords:** reformulation, clean label, obesity, glycaemic response, food industry, healthy

## Abstract

This study demonstrates a scenario of industrial reformulation by developing muffins that resemble store-branded ones and testing the possibility of reformulating them using inulin and green banana flour (GBF). Ten different formulations were created through reducing 10% or 30% of sugar and/or fat. Physical characteristics, consumer acceptance and purchase preferences, baking losses, nutritional properties, shelf-life, as well as cost and industrial processability were considered and discussed. Results on physical properties showed that firmness had increased in reformulated muffins while springiness only decreased when both sugar and fat were reduced by 30% (*p* < 0.05). Texture and sensory properties of reformulated muffins were acceptable, and the purchase intent rate was high. Regarding the nutritional properties, muffins incorporating more than 10% of fibres allowed the addition of nutritional claims. The incremental area under the curve iAUC_120min_ of blood glucose in healthy adults (*n* = 13) was significantly lower than control after ingesting 30% reduced sugar or fat muffins using inulin (*p* < 0.01). The microbial profile was not affected by reformulation during storage at 25 °C for 10 days. This study concluded that there is a significant potential to industrially produce reduced sugar or fat muffins using inulin or GBF up to 30% without significantly deteriorating quality attributes.

## 1. Introduction

Obesity as a global public health crisis, is only a few decades old coinciding with the technological revolution that happened in the 20th century [1]. More than 50% of people’s calorie intake in developed countries is derived from consuming ultra-processed foods [2] and hence the current food system plays a role in population obesity [3]. To help reduce obesity rates, transnational companies have been recently reformulating their products [4]. However, many small and medium sized companies (SMEs) do not have the necessary practical information on reformulation due to their limited budget for research and development [5]. A systematic knowledge-base for food reformulation targeted to SMEs was recently created to guide the food industry through the reformulation process through considering quality assessment, consumer acceptability, nutritional profile, legislations and industrial processability of the reformulated food product [6]. The current study on muffin reformulation based its investigation according to the guidelines demonstrated in the mentioned knowledge-base. 

American-style sweet muffins are widely consumed, and their market is projected to grow globally because of the increased demand on snacking products [7]. In the UK for example, muffins are one of the top four most consumed processed snacks [8]. Muffins are generally energy dense >4 kcal/g and contain on average high levels of sugar >22.5 g/100 g and fat >17.5 g/100 g [9]. Increasingly, manufacturers are driven to improve the nutritional quality of food products to compete against the expanding availability of healthy substitute snacks and hence continue to gain/maintain market share [10]. Moreover, producers are progressively challenged with stricter governmental targets as global obesity and its related comorbidities continue to rise [11], with recent guidelines recommending that sugar and energy content in muffins should not exceed 27.9 g/100 g and 325 kcal/portion, respectively [12]. 

There is no legislation that controls the macronutrient distribution and flavours of muffins; hence it is quite flexible to reformulate/alter their recipes. However, there are two main considerations for muffin reformulation. Firstly, reformulation should only consider using ‘clean-label’ alternatives [13] avoiding artificial and high intensity sweeteners as their use is not permitted in bakery products according to European law [14,15]. Secondly, reducing sugar and/or fat from muffins could alter their quality and sensory characteristics. For example, sugar serves as a bulking agent and plays a role in moisture control, shelf-life stability, texture enhancement, flavour, gas holding, limiting starch swelling during baking and forming an attractive light brown colour [16,17,18,19]. In terms of fat, it plays a role in reducing crumb firmness, stabilising the air in batters and improving palatability [20,21].

Many research studies have investigated reformulation to improving the nutritional profile of muffins by reducing their content of wheat flour, sugar and fat [16,22,23]. For instance, fibres such as pecan nut expeller meal [22], waxy whole wheat flour [24] and resistant starch [25] have been used for wheat flour reduction. For sugar reduction, high intensity sweeteners (e.g., stevia) [16], polyols [26] and fructan [27] have been used. While dietary fibres [28], fruit [29] and oleogels [30] have been used for fat reduction. 

The main aim of wheat flour and sugar reduction is to help decrease the glycaemic index/load [16,31,32]. While reduction of fat helps to decrease the energy density because fat possesses a higher energy content (9 kcal/g) than carbohydrate and protein (4 kcal/g) [33]. 

Inulin is a dietary fibre (fructan) that has prebiotic properties and consists of fructose molecules joined by β(2-1) bonds forming a mixture of oligomers and polymers that often end with a glucose molecule [34]; its degree of polymerisation is between 2 and 60 monomeric units, with an average of 12 [16]. Short-chain inulin has a sweet taste, high solubility and low viscosity, allowing its use as a sugar and fat replacer in numerous processed food products without impacting the quality properties of these products [35]. In general, inulin is found in a variety of plants and mainly derived from chicory roots, artichokes, garlic and banana [16]. In muffins, inulin is a potential sugar and fat substitute that could be used to reduce up to 50% of the sucrose [16] and fat [21], while potentially maintaining properties that are compatible with manufacturability, consumer acceptance and required shelf life. 

Green banana flour has a high nutritional value because it contains resistant starch up to 58.5% and fibres up to 15.5%, as well as phenolic acids including anthocyanins [36]. The incorporation of green banana flour in the food industry is increasing as large quantities of the fruit can be rejected due to skin defects or lower grade produce [37]. In food reformulation, green banana flour has promising results as a substitute, as it has been used in fat-reduced sausages [38] and chicken nuggets [39]. Moreover, green banana flour has been used to partially replace cassava starch in fish and cassava crackers [40] and partially replace flour in bread [41].

Reformulation studies on muffins have mostly used recipes consisting of basic ingredients which do not represent modern industrial scale production (e.g., with the use of emulsifiers, humectants and texture enhancers) [42,43]. Moreover, studies have not been designed to evaluate the industrial applicability of the reformulated muffins, as they have not investigated the effect of reformulation on essential characteristics of the end-product [23,44]. The aim of this study was to explore the possibility of reducing sugar and fat in muffins by creating and reformulating an industrial recipe that has similar macronutrient distribution of store-branded/packaged muffins using two clean-label substitutes: inulin and green banana flour. Muffins were created and evaluated following the systematic guidelines of the ‘Reformulation Knowledge-Base’ developed by Harastani et al. (2020) [6]. This approach included undertaking recipe development incorporating the fibres, followed by determining the effect of reformulation on muffins’ physical and sensory characteristics, purchase intent, cooking losses, nutritional properties (i.e., calorie reduction, packaging claims, in-vivo glycaemic properties and hunger perception), shelf-life, cost and industrial insights. 

## 2. Materials and Methods

### 2.1. Recipe Development

A control industrial muffin recipe was defined by surveying the ingredients and nutritional information used in packaged store branded muffins sold in four leading UK supermarkets (Asda, Morrisons, Sainsbury’s and Tesco). Ingredients of in-store muffins included: wheat flour, sugar, water, eggs, rapeseed oil, humectant (glycerol), wheat or corn starch, whey powder, skimmed milk powder, emulsifiers (e.g., soy lecithin, mono and diglycerides of fatty acids and/or sodium stearoyl-2-lactylate), raising agents (diphosphates, sodium carbonate, potassium carbonate), acidity regulator (citric acid), salt, stabilisers (xanthan gum or carboxy methyl cellulose), preservative (potassium sorbate) and flavouring. The average of added sugar (sucrose) was 22.3 ± 3.5 g/100 g, fat 18.26 ± 2.4 g/100 g and the theoretical calories were 377 g ± 26/100 g. 

To produce muffins that are close in properties to packaged muffins, the ingredients list formulated in this study is shown in Table 1. 

### 2.2. Muffin Ingredients

Ten muffin formulations were made by replacing sugar and/or fat at different percentages with inulin and/or green banana flour (GBF). Self-raising wheat flour (Tesco^®^), granulated sugar (Silver Spoon^®^, UK), baking powder (Dr. Oetker^®^, UK), glycerine (Dr. Oetker^®^, UK) instant dried skimmed milk powder (Tesco^®^) and rapeseed oil (Goldenfields^®^, UK) were purchased from Tesco, UK. Unflavoured whey protein concentrate (80% protein) and inulin were purchased from Bulk Powders^®^, UK. Fresh eggs and lemons were purchased from Aldi, UK. Organic green banana flour (containing 38% resistant starch and 12% fibres) was purchased from Natural Evolution^®^, Australia. Citric acid, xanthan gum, cornflour starch and soy lecithin powder were ordered from Buy Whole Foods Online^®^. Potassium sorbate was purchased online from Special Ingredients LTD, UK. Upon arrival, dry ingredients were stored in air-tight containers and all ingredients were kept in a cool and dry place until use.

### 2.3. Muffins Formulations

Sugar and/or fat were partially replaced with inulin and/or GBF as illustrated in Table 2. 

### 2.4. Muffin Preparation

Preparation of muffins is illustrated in the steps shown in Table 3.

### 2.5. Muffin Physical Properties

#### 2.5.1. Muffin Texture

A Texture Analyser (TA.XT plus, Stable Micro Systems Ltd., Surrey, UK) synced with Exponent Lite 32 software (Stable Micro Systems) was used to determine the firmness and springiness of the muffins. The muffins’ crusts were removed to avoid any irregularity allowing the cylindrical-shaped crumbs with a diameter of 50 mm to be tested. Texture analysis was performed using a trigger force of 5 g at a speed of 1 mm/s. A force was applied to the crumb using a cylinder probe with a radius of 36 mm. The probe compresses the sample by 25% of its height and holds at this distance for 60 s then withdraws from the sample and returns to its starting position. 

#### 2.5.2. Colour

The crust and crumb colours were determined using a portable colourimeter (Lovibond^®^ LC 100, The Tintometer Limited, Amesbury, UK). Colours were expressed in values of L* (lightness/darkness), a* (redness/greenness) and b* (yellowness/blueness). The colourimeter was calibrated using an in-built standard white tile. Six readings were taken for each sample and measurements were performed in triplicate for each formulation. 

### 2.6. Baking Losses

Six weight measurements, on different samples, were taken for each formulation before and after baking. Losses were calculated as the following:

(1) $$Baking\;loss\;\%=100-\left(100\times \frac{weight\;after\;baking\;}{weight\;before\;baking}\right)$$

### 2.7. Sensory Properties, Purchase Intent and Price

The conduction of this experiment was approved by Loughborough University Ethics Sub-Committee R19-P118. In total, 60 panellists from Loughborough University students and staff participated in this study after consenting and confirming that they neither have allergies to the muffin’s ingredients nor a history of anaphylaxis. A day prior to the sensory testing, muffins were baked and kept in airtight containers overnight at room temperature. Testing 11 different formulations by each participant was not practical in one sitting, hence each participant tested six formulations which included the control, and tasting was done in two sittings. Samples were coded by three-digit random numbers and served in portions of 30 g. Participants were advised to eat at least half of each sample. Water and crackers were available to cleanse participants palates between samples. A 5-point hedonic scale ranging from 5 to 1 (where: 5 = like very much, 4 = like moderately, 3 = neither like nor dislike, 2 = dislike moderately and 1 = dislike very much) was used to score the muffins ‘crumb colour’, ‘crust colour’, ‘smell’, ‘texture’, ‘softness’, ‘moistness’, ‘swallowability’, ‘taste’ and ‘overall acceptability’. 

Volunteers were also asked if they would be willing to purchase muffins represented by each of the samples by choosing one of these four options: ‘yes, absolutely, I like it’, ‘yes, I might occasionally’, ‘Yes, but only if a positive health claim is indicated on packaging’ or ‘No’. Moreover, participants were asked to choose how much they would be willing to pay for a pack of four muffins of each of the sampled formulations if these muffins were proven to be healthier than standard ones (knowing that an average pack of supermarket muffins costs £1.18). The range of prices was ‘<£1.00′, ‘£1.00–£1.50′, ‘£1.51–£2.00′ or ‘£2.01–£2.50′. 

### 2.8. Nutritional Properties

#### 2.8.1. Energy Reduction

Energy content was determined according to Atwater factor system [45,46] using updated calorie values on food components from CoFID^®^ [47] and FoodData Central^®^ [48] adopting the following formula:

(2) $$\mathrm{Energy}\;\mathrm{content}=\overset{n}{\underset{1}{\sum }}\left[(m_{1}\times E_{d1})+\left(m_{2}\times E_{d2}\right)+\ldots \right]$$

where *m_n_* is the mass (g) of ingredient *n* and *E_dn_* is the energy density of ingredient *n*. Calculations considered that inulin provides 2.10 kcal/g and GBF 3.38 kcal/g as reported by the suppliers. 

#### 2.8.2. Glycaemic Response

##### Study Design

This experiment was approved by Loughborough University Ethics Sub-Committee R19-P119. A total of 15 human subjects participated in this randomised, controlled, crossover and single-blind intervention study. The study design and protocol recommendations were adopted from Soong et al. (2015) [31], Stewart and Zimmer (2018) [32] and Brouns et al. (2005) [49]. The inclusion criteria included healthy female and male adults (18–60) years old, with a body mass index of between 18.0 and 29.9 kg/m^2^, who have a fasting blood glucose < 5.5 mmol/L. Females were on oral contraceptives or tested in the luteal phase of the menstrual cycle (between day 14–28 after the onset of menses). Exclusion criteria included being diagnosed with a metabolic or chronic disease (e.g., type 2 diabetes, cancer or chronic gastrointestinal problems), using medications to control blood glucose, pressure and cholesterol, being sensitive or allergic to muffins ingredients and if female, being pregnant or breastfeeding. 

Participants completed a food and exercise diary for the day before the first trial and followed the same diet and physical activity pattern avoiding any high intensity training before each visit. They were also asked to keep activity to minimum in the morning of the testing day. Participants arrived at 09:00 after a 12-h overnight fast on three visits to ingest a muffin on each visit (i.e., control, 30_Sugar_Inulin or 30_Fat_Inulin). Muffin formulations were randomised to control for systematic ‘order’ effects. Upon arrival and after consenting, height and weight were measured, and a health screening questionnaire was given to ensure participants meet the inclusion criteria. Capillary blood samples were collected at a fasting state, then each participant consumed 106 g of the specified muffin (equivalent to 50 g of available carbohydrates considering an approximate 8% water loss during baking) within five minutes. Blood was then collected at 15, 30, 45, 60, 90 and 120 min after eating the muffins. During testing, participants rested quietly, reading or using their laptops. Trials were separated by at least 2 days. 

##### Blood Collection and Analysis

Capillary blood samples (20 µL) were collected by finger prick using a single-use lancet device (Accu-Chek^®^ Safe-T-Pro Plus, Roche, UK). Blood glucose was measured using a Biosen C-Line Glucose and Lactate Analyser after calibration. The incremental area under the curve iAUC was calculated ignoring the area under the baseline using the trapezoidal rule as described by Brouns et al. (2005) [49]. 

#### 2.8.3. Hunger

During participation in the glycaemic response study, the same volunteers were given a sheet that contained a hunger scale. Participants expressed their subjective hunger level by indicating on a 10 cm long scale that ranged from ‘not hungry at all’ to ‘extremely hungry’ at different times: pre-muffin, immediately after muffin consumption, then at 30, 60, 90 and 120 min after eating the muffin. The incremental area under the curve iAUC was calculated using the trapezoidal rule as described by Quilez et al. (2007) [50]. 

### 2.9. Muffins Shelf-Life 

From a microbial perspective, the shelf-life of the three muffin formulations that were tested for their glycaemic response (control, 30_Sugar_Inulin and 30_Fat_Inulin) was investigated. After baking and cooling, muffins were packed in clean transparent and sealable bags. Each bag contained 3 muffins of the same formulation to allow testing in triplicates. Muffins were stored at 25 °C and testing was done using the pour-plate method on day 1, 3, 7 and 10 of storage to determine the total viable counts using Nutrient Agar (Thermo Fisher Scientific Oxoid Ltd., UK) and yeasts and moulds using Potato Dextrose Agar (Thermo Fisher Scientific Oxoid Ltd., UK). Nutrient Agar plates were incubated at 37 °C and counted after 24 h, while Potato Dextrose Agar plates were incubated at 25 °C and counted after 48 h. 

### 2.10. Statistical Analysis 

In terms of the texture and sensory data, analysis of variance (One-Way ANOVA) was performed using IBM SPSS Statistics 24. A Post-Hoc was applied to consider the Least Significant Differences and Tukey’s test at a significance of *p* < 0.05.

In terms of the glycaemic response and hunger data, iAUC results were analysed by repeated measures ANOVA using compare main effects (Pairwise comparisons at a significance of *p* < 0.05).

## 3. Results and Discussion

### 3.1. Muffin Texture Properties 

**Figure 1:**
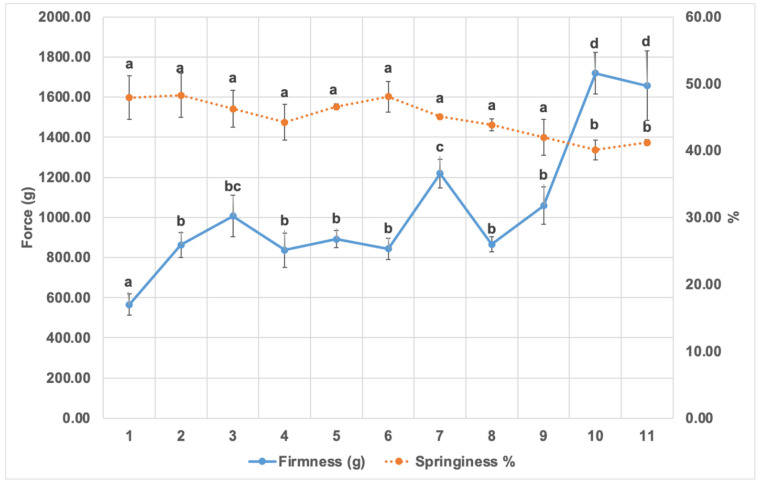
The firmness (g) and springiness (%) of different muffin formulations. Means with different letters are significantly different (*p* <0.05). Where: (1) control (2) 10_Sugar_Inulin (3) 30_Sugar_Inulin (4) 10_Fat_Inulin (5) 10_Fat_GBF (6) 30_Fat_Inulin (7) 30_Fat_GBF (8) 10_Sugar_Inulin_10_Fat_Inulin (9) 10_Sugar_Inulin_10_Fat_GBF (10) 30_Sugar_Inulin_30_Fat_Inulin (11) 30_Sugar_Inulin_30_Fat_GBF.

Texture results represented by firmness and springiness are illustrated in Figure 1. Results show that control muffins were significantly less firm than all reduced sugar and/or fat formulations (*p* < 0.05). Results also show that the firmness was significantly affected by the amount of sugar/fat reduction and the type of fibres added. Reduction of sugar and/or fat by 10% showed less firmness than that of 30% reduction. Moreover, at 30% reduction, fat reduced muffins with inulin (30_Fat_Inulin) had less firmness than those reduced with green banana flour (30_Fat_GBF) which could be due to the higher solubility and smaller molecular structure of inulin fibres. The reduction of both sugar and fat by 30% resulted in the highest firmness levels compared to the control. These findings agree with those of Zahn et al. (2010) [21] who found that replacing 50% of fat with inulin in muffins resulted in higher firmness and with Struck et al. (2016) [43] who also reported higher firmness in 30% reduced sugar muffins by dietary fibres. This is because fibres possess higher water binding capacity which reduces the water available for other ingredients such as the starch and hence affects product characteristics [51]. Gao et al. (2016) [16] reported increased firmness of muffins when sugar was fully replaced with inulin or Stevianna but found that firmness was not affected when sugar was partially replaced by 50%. 

As shown in Figure 1, muffin springiness was not influenced significantly with sugar/fat reduction except in two samples: 30_Sugar_Inulin_30_Fat_Inulin and 30_Sugar_Inulin_30_Fat_GBF where it was lower than control reflecting a more compact crumb texture. Our findings are comparable with Zahn et al. (2013) [27], Rodriguez-Garcia et al., (2014) [34] and Martinez-Cervera et al. (2012) [52] who also found that springiness decreases when fibre content is highly increased. That is because sugar contributes to a soft and springy texture by increasing the temperature of starch gelatinisation [16] and elevating that of egg white protein denaturation during baking which helps in air cell and volume formation [34,53]. Fat increases the volume and softness of muffins by disrupting the gluten network structure of wheat flour and by stabilising the air bubbles due to their surface-active properties [54]. 

### 3.2. Colour

Crust and crumb colour of muffin formulations are addressed as L*, a* and b* values in Table 4. Formulations that involved sugar and/or fat substitution with inulin had crust and crumb colour parameters that are not significantly different from control. Similarly, formulations incorporating GBF at 10% had colour characteristics that resemble control muffins. However, formulations that included 30% GBF as a fat substitute had a darker crust than all other muffins represented with lower b* (yellowness) values (*p* < 0.05). Moreover, the addition of 30% GBF resulted in darker crumbs including lower L* (lightness) but higher a* (redness) values (*p* < 0.05). The difference in muffin colour is probably due, at least in part, to the initial colour of raw materials used as inulin had a white-creamy colour and GBF had a brownish colour. Changes in crumb colour of reformulated cakes and muffins using dietary fibres were also reported by Zahn et al. (2010) [21], Sanz et al. (2009) [25], and Heo et al. (2019) [55]. 

### 3.3. Baking Losses

Determining the percentage of moisture loss is important for manufacturers because it affects the weight and water activity of the end-product. In this study, baking losses maintained a value between 7.31% and 9.14% probably due to presence of the texture-enhancing ingredients (i.e., lecithin, xanthan gum and glycerine). As shown in Table 5, muffins that contained the highest fibre content had the least baking losses, that is likely because of the higher water binding capacity of these fibres (*p* < 0.05). Similarly, Heo et al. (2019) [55] reported a decreased baking loss rate by the addition of kimchi fibres into muffins. Moreover, increased moisture was reported by Martinez-Cervera et al. (2011) [56] in muffins stored for 24 h in which part of the fat was replaced with cocoa fibre. 

### 3.4. Sensory Analysis and Purchase Intent

#### 3.4.1. Sensory Properties

**Figure 2:**
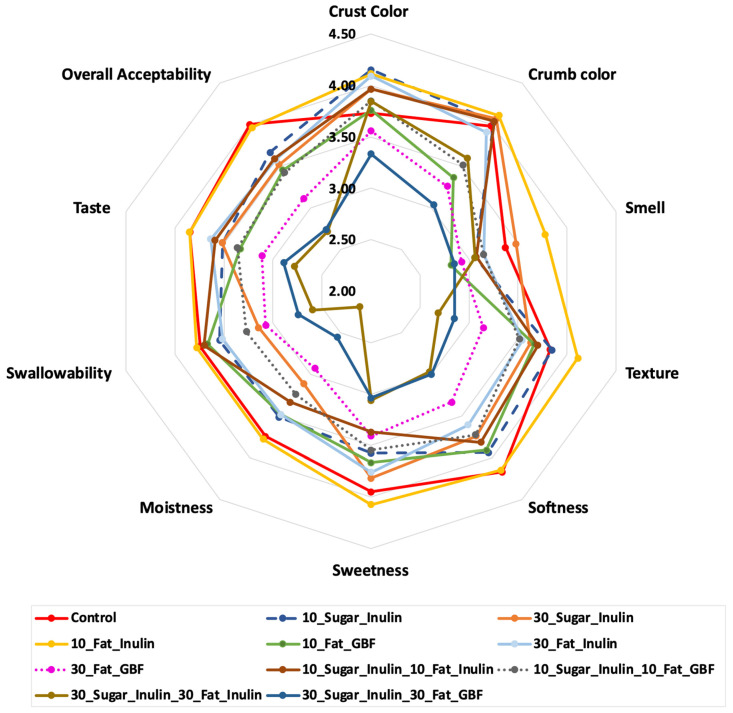
Sensory Properties of Muffin Formulations (*n* = 60).

The sensory properties for muffins including ‘crust colour’, ‘crumb colour’, ‘smell’, ‘texture’, ‘softness’, ‘sweetness’, ‘moistness’, ‘swallowability’, ‘taste’ and ‘overall acceptability’ are shown in Figure 2. Statistical analysis of sensory values showed that there was no significant difference of consumer preferences between reformulated muffins and the control in terms of the crust colour.

**Figure 3:**
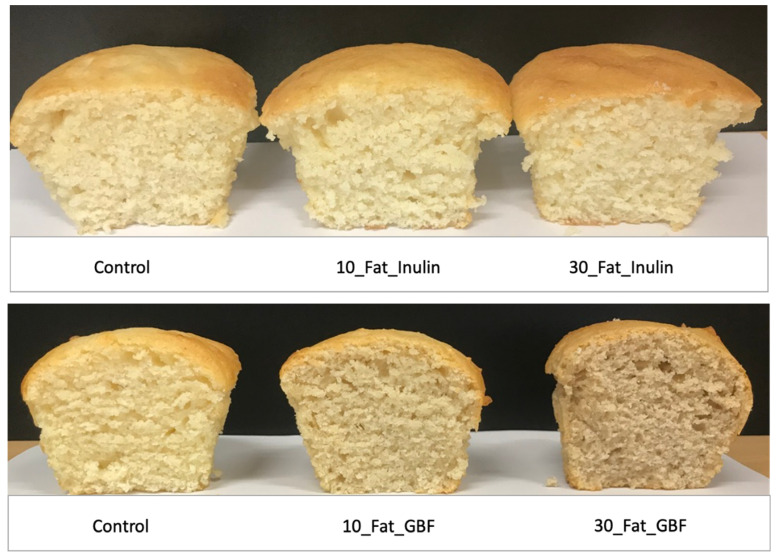
Formulations of fat reduced muffin using inulin or GBF.

However, mean values of crumb colour were significantly less compared to control in samples containing GBF (i.e., 10_Fat_GBF, 30_Fat_GBF and 30_Sugar_Inulin_30_Fat_GBF) due to the darker colour of incorporated fibres (see Figure 3). No significant differences were reported for smell, but texture and softness scored lower in samples that contained 30% GBF and the ones with the highest fibre content including 30_Sugar_Inulin_30_Fat_Inulin and 30_Sugar_Inulin_30_Fat_GBF due to the significant firmer texture of these formulations. Sweetness was not significantly influenced by sugar reduction in 10_Sugar_Inulin, 30_Sugar_Inulin or 10_Sugar_Inulin_10_Fat_Inulin and 10_Sugar_Inulin_10_Fat_GBF possibly indicating that sugar is added in excessive amounts in commercial muffins and highlighting a considerable margin of reduction. Mean values of moistness, swallowability, taste and overall acceptability were comparable with control even at 30% reduction of sugar or fat using inulin. However, slightly decreased values in these properties were found in 30_Fat_GBF, 30_Sugar_Inulin_30_Fat_Inulin and 30_Sugar_Inulin_30_Fat_GBF (*p* < 0.05). In conclusion, panellists showed compatible overall acceptability with control in seven out of ten reduced sugar and/or fat formulations including 10_Sugar_Inulin, 30_Sugar_Inulin, 10_Fat_Inulin, 10_Fat_GBF, 30_Fat_Inulin, 10_Sugar_Inulin_10_Fat_Inulin and 10_Sugar_Inulin_10_Fat_GBF. Acceptable cakes that had a replacement of 30% sugar and/or 50% fat by inulin were also reported by Rodriguez-Garcia et al. (2014) [34]. 

#### 3.4.2. Purchase Intent and Price

**Figure 4:**
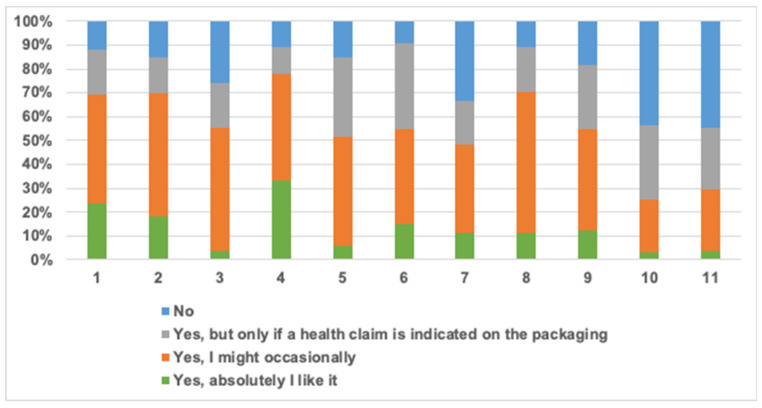
Purchase intent of panellists of muffin formulations (*n* = 60). Where: (1) control (2) 10_Sugar_Inulin (3) 30_Sugar_Inulin (4) 10_Fat_Inulin (5) 10_Fat_GBF (6) 30_Fat_Inulin (7) 30_Fat_GBF (8) 10_Sugar_Inulin_10_Fat_Inulin (9) 10_Sugar_Inulin_10_Fat_GBF (10) 30_Sugar_Inulin_30_Fat_Inulin (11) 30_Sugar_Inulin_30_Fat_GBF.

Figure 4 illustrates the intent of purchase ‘Yes, I absolutely like it’, ‘Yes, I might occasionally’, ‘Yes, but only if a health claim is indicated on the packaging’ and ‘No’. Results show a high purchase intent ranging from 55.6% to 90.9% in all muffin formulations.

The formulation 10_Fat_Inulin and control muffins had the highest percentage ‘Yes, absolutely I like it’ response at 33.3% and 27.7% respectively. Sugar reduced muffins by inulin at 30% had a positive purchase intent of 74.1% including 3.7% ‘Yes, absolutely I like it’, 51.9% ‘Yes I might occasionally’ and 18.5% ‘Yes, but only if a health claim is indicated on the packaging’ which emphasises the possibility of using inulin as a sugar replacer in commercial muffins. The formulation of 30_Fat_Inulin had the highest total positive purchase intent at 90.9%, followed by 10_Sugar_Inulin_10_Fat_Inulin at 88.9%, 10_Fat_Inulin at 88.8% surpassing that of control at 88.1%. 

Formulations that had the highest fibre content, least sensory scores and firmest texture values had the highest percentage of ‘No’ as a response for the intent of purchase represented by 43.8% and 44.4% for 30_Sugar_Inulin_30_Fat_Inulin and 30_Sugar_Inulin_30_Fat_GBF respectively. Moreover, formulations that had inulin as a fat replacer were generally more preferred than the ones containing GBF. This could be due to the differences of colour and sweetness of these fibres which could be overcome/masked by adding common muffin flavourings such as blueberries, cocoa and/or chocolate. 

**Figure 5:**
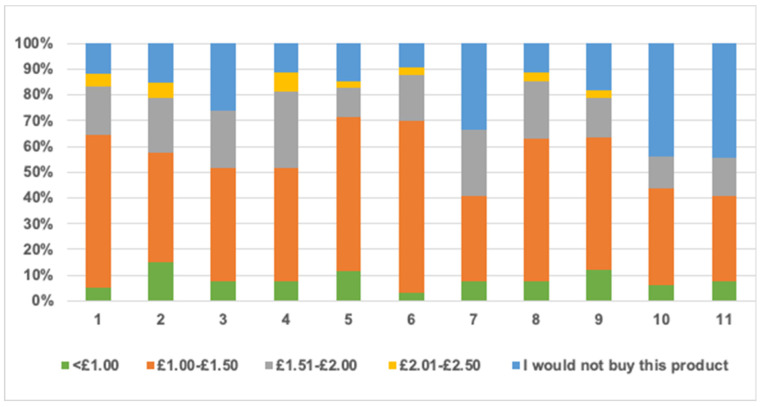
The maximum price that panellists were willing to pay for muffin formulations (*n* = 60). Where: (1) control (2) 10_Sugar_Inulin (3) 30_Sugar_Inulin (4) 10_Fat_Inulin (5) 10_Fat_GBF (6) 30_Fat_Inulin (7) 30_Fat_GBF (8) 10_Sugar_Inulin_10_Fat_Inulin (9) 10_Sugar_Inulin_10_Fat_GBF (10) 30_Sugar_Inulin_30_Fat_Inulin (11) 30_Sugar_Inulin_30_Fat_GBF.

Regarding muffins price, generally, the price range of £1.00–£1.50 had the highest votes for all formulations followed by £1.51–£2.00 and <£1 while the most expensive price range £2.01–£2.50 had the lowest votes. Figure 5 shows that participants valued formulations that had a 10% reduction of sugar and/or fat at a higher price than those with a 30% reduction. This pattern was consistent and reliable in all formulations. 

### 3.5. Nutritional Properties

#### 3.5.1. Calorie Reduction and Nutritional Claims

The theoretical energy content in muffin formulations, possible nutritional claims as well as traffic light colours for sugar and fat are presented in Table 6. Reduction of sugar by 10% and 30% using inulin decreased the energy content by 1.02% and 3.06%, respectively. A phrase of ‘Reduced sugar—High in fibre’ can be claimed on 30_Sugar_Inulin packaging according to the European Commission [57]. As expected, a more profound reduction in energy by 7.36% and 9.08% is found in reduced fat muffins 30_Fat_GBF and 30_Fat_Inulin respectively where a claim of ‘Reduced fat—A source of fibre’ is possible on packaging. Formulations of 10_Sugar_Inulin_10_Fat_GBF and 10_Sugar_Inulin_10_Fat_Inulin can be marketed as ‘A source of fibre’ as they contain ≥3 g of fibre/100 g. The most significant decrease in energy was in 30_Sugar_Inulin_30_Fat_GBF and 30_Sugar_Inulin_30_Fat_Inulin muffins achieving a reduction of 10.42% and 12.14% respectively and a possible marketing claim of ‘Reduced sugar—Reduced fat—High in fibre’. 

In terms of traffic light colouring system on the Front of Pack FoP, according to FSA (2016) [58], control muffins had a red traffic light colour (>22.5 g/100 g) for sugar and an amber one (>3.0 to ≤17.5 g/100 g) for fat. The red colour of sugar had shifted to amber in reduced sugar muffins, while the one for fat stayed in the amber range in all formulations even those that involved fat reduction. 

Testing the in-vivo glycaemic potency of all muffin formulations was not practical because it would mean that volunteers must commit to 11 testing sessions and hence participant recruitment, compliance and completion of study were realistic barriers to obtaining meaningful results. Consequently, after careful consideration of previously presented results, it was decided to test the glycaemic response of 30_Sugar_Inulin and 30_Fat_Inulin and compare it to control as these two reformulated muffins showed positive physical characteristics, sensory properties and high purchase intent as well as the ability to be commercialised and marketed under a positive nutritional claim. 

#### 3.5.2. Glycaemic Response 

The 15 volunteers that met the inclusion criteria were initially recruited to perform the glycaemic response trial. One participant dropped out after one visit due to reporting a digestive intolerance to inulin. Another participant was excluded due to doing a high intensity exercise before one testing session. Consequently, the study collected and analysed the data of 13 participants (Table 7). 

**Figure 6:**
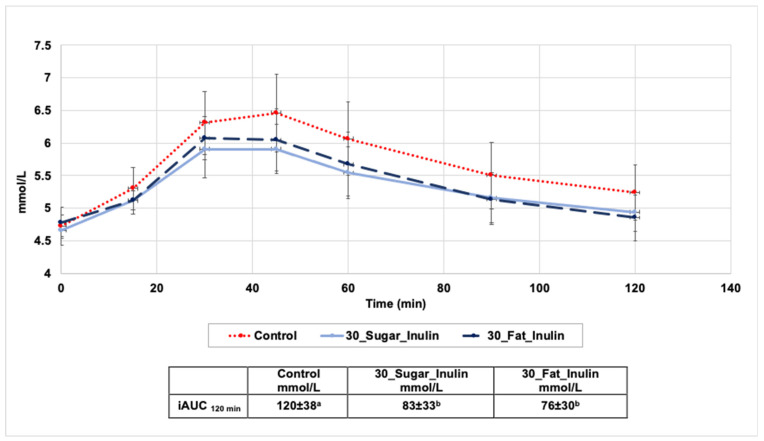
Mean blood glucose responses for control, 30_Sugar_Inulin and 30_Fat_Inulin. Means with different letters are significantly different *p* < 0.01.

The iAUC (Figure 6) of glucose was significantly lower in 30_Sugar_Inulin and 30_Fat Inulin compared to control (F:1-12 = 129, *p* < 0.01). The mean glucose peak of control muffin was 6.46 ± 0.59 mmol/L, higher than 5.91 ± 0.44 mmol/L and 6.07 ± 0.33 mmol/L for 30_Sugar_Inulin and 30_Fat_Inulin, respectively. After 2 hours of muffins ingestion, the return to fasting blood glucose levels was faster with the reformulated muffins. Pairwise comparisons indicate no significant difference between iAUC of 30_Sugar_Inulin and 30_Fat_Inulin. The reduction of postprandial glucose between individuals may be attributed to the presence of soluble fibres that can slow the release of glucose by increasing the viscosity of the gut digesta and limit its interaction with digestive enzymes [59]. Soluble fibres can also reduce the postprandial glucose by slowing gastric emptying and decreasing the accessibility of a-amylase to the substrate (starch) [60]. Brennan et al. (2004) [61] found that adding inulin to pasta at (2.5 g, 5.0 g, 7.5 g, 10.0 g/100 g flour) had reduced the predicted glycaemic index (in vitro) by 2.3–15%. Borczak et al., (2012) [62] found that a significant 35% reduction in in vivo glycaemic index of bread rolls was possible when inulin (added at 2.5%) was coupled with a freezing process of these rolls (−18 °C for 14 days) where resistant starch type3 was allowed to form. Moreover, Lightowler et al. (2018) [63] found a linear correlation (r = 0.990, *p* < 0.001) between post prandial glucose reduction and inulin-type fructans when used in substitution of sugar in several foods and drinks. In fact, the latter found that reduction of 30% sugar in yogurt and fruit jellies using inulin had decreased the total iAUC_120 min_ by 14% and 16%, respectively. 

#### 3.5.3. Hunger Perception

**Figure 7:**
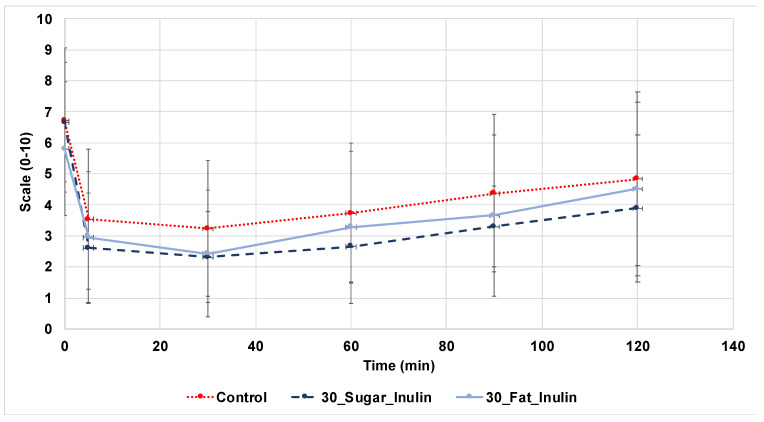
Subjective hunger score of 13 participants for Control, 30_Sugar_Inulin and 30_Fat_Inulin muffins.

Perception of hunger illustrated in Figure 7 indicates that 30_Sugar_Inulin and 30_Fat_Inulin had a higher satiety effect than the control by projecting a decreased mean of iAUC_120 min_, however, these values are not significant. Hunger values were not homogeneous due to the subjective type of data collected and the small number of participants in this trial. Addition of viscous fibres to diet generally decreases feelings of hunger due to inducing satiety, but there are inconsistent reports of the satiety effect of inulin due to its high solubility and low viscosity [64]. For example, reduced fat sausages that were reformulated by adding 24 g of inulin had no significant satiety effect from control sausages [65]. However, recent studies show that consuming a diet that contains inulin-rich vegetables or inulin supplements is linked to a higher satiety and fullness feeling, and a decreased hunger and desire to eat fatty, sugary and salty foods [66,67]. 

### 3.6. Shelf-Life

From a microbial point of view, the shelf-life of aerobically stored cakes/muffins is mostly determined by investigating the growth of spore forming mesophilic aerobic bacteria (e.g., Bacillaceae) and yeasts and moulds [68,69]. The baking process of cakes destroys most microbial spores. However, some spores might survive [70] and post-baking contamination might occur during cooling and packaging [71]. In this study, the shelf-life of control, 30_Sugar_Inulin and 30_Fat_Inulin muffins was assessed. During 10 days of storage at 25 °C, growth of yeasts and moulds was below the limit of detection in all samples <1 log CFU/g (see Table 8). That is anticipated due to the presence of potassium sorbate (added at 0.04 g/100 g; pH of muffins was 6.5 ± 0.1) which is effective at this concentration against yeasts and moulds [68]. Without the use of preservatives, cakes might have a shorter shelf-life; Rodriguez et al. (2002) [69] found that their cake samples had a mouldy surface after the sixth day of storage at 15–20 °C when no preservative was used. 

In this study, the total viable counts were below the limit of detection for all samples in the first day of storage. A few bacterial colonies were detected on the third and seventh day of storage as some spores might have vegetated but could not survive to reach the tenth day of storage as shown in Table 8. This is most likely due to the presence of potassium sorbate. Addition of inulin in 30_Sugar_Inulin and 30_Fat_Inulin did not affect muffins shelf-life negatively as the counts of bacterial colonies in reformulated muffins were comparable with control at ~1 log CFU/g even at the peak i.e., on days 3 and 7. This is at least 3 orders of magnitude less than the minimum bacterial counts associated with food-borne illnesses > 4–5 log CFU/g [68,72]. 

## 4. Additional Costs and Industrial Considerations

Estimated additional costs of reformulated muffins are shown in Table 9 considering that the retail costs of inulin, GBF, rapeseed oil and sugar were £23/kg, £22/kg, £2/L and £1/kg respectively. Results show that addition of raw materials (i.e., inulin and/or GBF) elevated the average price of original muffins. The study had addressed previously that the majority of participants (see Figure 5) preferred to purchase the muffins formulations at a price ranging from £1.00–£1.50. From the data presented in Table 9, it is found that the cost of reformulated muffins either falls in this range when sugar and/or fat was reduced by 10%, or slightly exceeds it when sugar or fat was reduced by 30% (i.e., 30_Sugar_Inulin, 30_Fat_Inulin and 30_Fat_GBF). However, the cost rose to £2.00 and £2.02 when both sugar and fat were substituted with fibres at 30%. The amounts of elevated costs are not conclusive in this study, because raw materials were purchased in small amounts where retail prices are probably higher than prices of bulk quantities when purchased by industrial enterprises.

There are no legislations that prevent the use of inulin or GBF in muffins and both are considered as plant based (dietary fibres) ‘clean label’ substitutes. However, inulin doses over 15–30 g/d were associated with reports on slight gastrointestinal discomforts including bloating, changes in stool consistency and frequency [73,74]. Bonnema et al. (2010) [75] reported that consuming inulin at a dose up to 10 g/day was not associated with adverse effects in healthy adults. In the current study, inulin was used up to 9.7 g/portion (see Table 6), which does not exceed the recommended tolerable dose. 

To make these muffins suitable for the industrial production, large scale suppliers for inulin and/or GBF would need to be identified. Reproducibility and quality control would need to be key considerations of further development of this work.

From a processing perspective, as fibres increase the viscosity of muffin batters [43], production parameters may need to change, e.g., higher energy settings might be required during batter mixing and through pumping and dosing into paper cups, which could also add to production costs. For commercial production, modifications or upgrades of production equipment may be required to achieve the desired quality and throughput of muffin production. 

It is worth highlighting that despite the likelihood of increased cost, companies that take responsibility towards improving the nutritional profile of their products, probably have a higher chance of market competitiveness and success because of increased consumer trust and loyalty towards them [4,76]. 

## 5. Conclusions

This paper presented a novel approach to muffin reformulation by developing an industrial recipe that mimics the portion size and macronutrient distribution of in-store muffins and then reformulating this to explore the suitability of different, healthier, ingredient substitutions. The reformulation process was assessed holistically through studying the quality, consumer acceptability, purchase intent, nutritional properties and shelf-life of the reformulated muffins, as well as, considering their suitability to be manufactured on an industrial scale. 

Results showed that muffin firmness increased in all samples that incorporated inulin and/or GBF (*p* < 0.05) and the values of this increase were positively correlated with the increased amount of fibre content. Springiness was only affected by reformulation when fat and sugar were both substituted with fibres at 30% (*p* < 0.05). Muffin colour was not affected by reformulation in samples containing inulin. However, when fat was reduced by 30% using GBF, muffins had darker crusts and crumbs due to the original colour of the fibres added. Adding inulin and/or GBF to the muffins slightly decreased the baking losses due to the higher water binding capacity of these fibres. Sensory analysis showed that participants (*n* = 60) expressed high values of overall acceptance to most reformulated muffins. However, participants were able to detect samples that had 30% of both sugar and fat reduction and scored them significantly lower than control samples. Moreover, sensory properties of samples containing 30% GBF were also lower than control probably due to their apparent darker colour that might be perceived as ‘a healthier option’ which is linked generally to lower sensory preferences. Regarding the intent of purchase, impressively, muffins had a positive purchase intent between 74% and 90.9%, except for 30_Fat_GBF (66.7%) 30_Sugar_Inulin_30_Fat_Inulin (56.3%) and 30_Sugar_Inulin_30_Fat_GBF (55.6%). The most frequent answer for the intent of purchase was ‘yes, I might buy them occasionally’ at a price range of ‘£1.00–£1.50′. 

From a nutritional point of view, the use of fibres had reduced the calories in the reformulated muffins in a range from 1.02% to 12.14%, with the more profound reductions when fat was reduced. The incorporation of fibres in muffins allowed variable nutritional claims to be possibly added on packaging except for 10_Sugar_Inulin, 10_Fat_Inulin and 10_Fat_GBF because the amount of fibre added in these samples was <3 g/100 g. The traffic light colour that could be present on the front of muffins package shifted from red to amber in reduced sugar muffins, while the one for fat stayed amber in all formulations, including those that involved fat reduction. 

Testing the glycaemic response of control, 30_Sugar_Inulin (containing 7.9 g inulin/106 g muffins) and 30_Fat_Inulin (containing 5.8 g inulin/106 g muffins) in 13 healthy human volunteers showed significant decrease in the iAUC_120 min_, but responses on satiety were not conclusive. The microbial profile of 30_Sugar_Inulin and 30_Fat_Inulin tested during storage at 25 °C for 10 days showed no significant difference from control. After evaluation of previous results and consideration of legislations, acceptable daily intakes and potential business considerations, this study found that it is possible to produce reduced sugar or fat muffins using inulin up to 30% on an industrial scale. It is also possible to use GBF up to 30% for fat reduction, preferably with the addition of other ingredients, such as cocoa or fruits, to improve the colour of the final product. 

## Figures and Tables

**Table 1:** Composition of the control muffin mimicking industrial muffin ingredients.

Ingredient	Mass (g)	g/100 g	Baker’s Mass *%
Flour	125	25.95	100
Sugar	110	22.84	88.00
Rapeseed oil	80	16.61	64.00
Tap water	70	14.53	56.00
Eggs	60	12.46	48.00
Starch	15	3.11	12.00
Skimmed milk powder	8	1.66	6.40
Baking powder	2.5	0.52	2.00
Glycerine	2.5	0.52	2.00
Soy lecithin	2.5	0.52	2.00
Whey protein powder	2	0.42	1.60
Lemon peel (Flavouring)	2	0.42	1.60
Citric acid	1	0.21	0.80
Xanthan gum	1	0.21	0.80
Potassium sorbate	0.2	0.04	0.16
Batter total weight	481.7	100	385.36

* Baker’s mass is calculated based on the flour%.

**Table 2:** Formulations experimented to produce sugar and/or fat reduced muffins.

	Formulation	Formulation Code	Sugar *	Rapeseed Oil *	Inulin *	GBF *
Original muffins	(1) Control	Control	110	80	-	-
Sugar reduced muffins	(2) 10% reduced sugar using inulin	10_Sugar_Inulin	99	80	11	-
	(3) 30% reduced sugar using inulin	30_Sugar_Inulin	77	80	33	-
Fat reduced muffins	(4) 10% reduced fat using inulin	10_Fat_Inulin	110	72	8	-
	(5) 10% reduced fat using GBF	10_Fat_GBF	110	72	-	8
	(6) 30% reduced fat using inulin	30_Fat_Inulin	110	56	24	-
	(7) 30% reduced fat using GBF	30_Fat_GBF	110	56	-	24
Sugar and fat reduced muffins	(8) 10% reduced sugar and 10% reduced fat using inulin	10_Sugar_Inulin_10_Fat_Inulin	99	72	19	-
	(9) 10% reduced sugar using inulin and 10% reduced fat using GBF	10_Sugar_Inulin_10_Fat_GBF	99	72	11	8
	(10) 30% reduced sugar and 30% reduced fat using inulin	30_Sugar_Inulin_30_Fat_Inulin	77	56	57	-
	(11) 30% reduced sugar using inulin and 30% reduced fat using GBF	30_Sugar_Inulin_30_Fat_GBF	77	56	33	24

* Values presented are in grams based on the batter’s total weight illustrated in Table 1.

**Table 3:** Muffin preparation.

Step	Method
(1) Preparing the emulsification gel and preservative mixture	(a) Lecithin was added to 50 mL water and mixed at speed 3 (high speed) for 2 min (VonShef model 07/067 hand mixer). (b) Xanthan gum and potassium sorbate were mixed with glycerine manually. (a) and (b) were added and mixed at speed 1 (low speed) for 1 min.
(2) Mixing	Sugar was mixed with eggs, oil and lemon peel at speed 2 (moderate speed) for 2 min until a creamy colour was formed.
(3) Preparing the acidity regulator	Citric acid was dissolved in 20 mL water.
(4) Mixing dry ingredients	Wheat flour, corn starch, whey, skimmed milk powder and baking powder were sifted and mixed manually with a whisk. Inulin and GBF were added in this step when they were used as sugar/fat substitutes.
(5) Combining	Components of step (2) and (3) were mixed at low speed for 30 s then mixed with components of step (1).
(6) Final muffin batter	Components of step (4) were mixed gradually with components in step (5) at low speed for 1 min.
(7) Filling and baking	The resulting batter was filled into muffin paper cups each weighing 82 ± 2 g. The muffin tray was then placed on the middle rack of an electric fan oven (John Lewis & Partners JLBIOS616 Single Oven, Stainless Steel) which was set to 180 °C (top and bottom) for 22 min.
(8) Cooling	Baked muffins were left to cool on a cooling rack for 60 min then packed in clean airtight bags for further analysis.

**Table 4:** Crust and crumb colour of muffin formulations.

Formulation	Crust			Crumb		
	L*	a*	b*	L*	a*	b*
Control	60.20 ± 3.15	11.50 ± 3.91	33.27 ± 2.14	72.87 ± 2.37	0.43 ± 0.40	18.57 ± 0.90
10_Sugar_Inulin	60.06 ± 5.84	10.16 ± 5.41	33.54 ± 2.28	74.35 ± 0.70	0.33 ± 0.10	16.03 ± 0.65
30_Sugar_Inulin	55.80 ± 1.21	16.87 ± 1.10	33.47 ± 1.23	75.63 ± 0.87	0.37 ± 0.31	17.63 ± 0.97
10_Fat_Inulin	54.87 ± 4.50	16.49 ± 2.63	33.03 ± 2.50	74.20 ± 1.44	0.17 ± 0.15	15.63 ± 2.02
10_Fat_GBF	57.28 ± 4.72	13.08 ± 2.85	29.88 ± 1.01	69.92 ± 2.58	2.06 ± 1.56	16.93 ± 1.46
30_Fat_Inulin	61.88 ± 3.50	10.80 ± 4.43	33.45 ± 2.96	74.23 ± 1.46	0.98 ± 0.59	16.50 ± 1.06
30_Fat_GBF	54.70 ± 2.16	15.47 ± 0.58	26.77 ± 2.38 ^a^	66.47 ± 2.11 ^a^	4.23 ± 0.55 ^a^	18.47 ± 0.38
10_Sugar_Inulin_10_Fat_Inulin	59.20 ± 1.38	14.30 ± 0.73	35.40 ± 1.27	74.38 ± 1.51	0.50 ± 0.12	16.28 ± 0.78
10_Sugar_Inulin_10_Fat_GBF	54.80 ± 2.26	15.48 ± 1.09	33.53 ± 1.73	72.23 ± 3.16	1.80 ± 0.72	16.47 ± 0.95
30_Sugar_Inulin_30_Fat_Inulin	54.42 ± 4.92	17.48 ± 1.71	30.40 ± 2.06	76.30 ± 0.35	0.60 ± 0.20	16.40 ± 0.53
30_Sugar_Inulin_30_Fat_GBF	57.28 ± 0.94	14.23 ± 1.98	28.48 ± 2.21 ^a^	65.73 ± 0.67 ^a^	4.20 ± 0.10 ^a^	18.00 ± 0.44

Means within the same column marked with the letter ^a^ are significantly different at *p* < 0.05.

**Table 5:** Baking loss of muffin formulations.

Formulation	Mean Percentage of Baking Loss%
Control	9.14 ± 0.35 ^a^
10_Sugar_Inulin	8.72 ± 0.40 ^a^
30_Sugar_Inulin	8.86 ± 0.49 ^a^
10_Fat_Inulin	8.52 ± 0.30 ^a^
10_Fat_GBF	8.60 ± 0.36 ^a^
30_Fat_Inulin	8.63 ± 0.33 ^a^
30_Fat_GBF	8.54 ± 0.49 ^a^
10_Sugar_Inulin_10_Fat_Inulin	9.03 ± 0.42 ^a^
10_Sugar_Inulin_10_Fat_GBF	7.78 ± 0.39 ^b^
30_Sugar_Inulin_30_Fat_Inulin	7.90 ± 0.35 ^b^
30_Sugar_Inulin_30_Fat_GBF	7.31 ± 0.30 ^b^

Means of percentage of baking loss with different letters are significantly different at *p* < 0.05.

**Table 6:** Theoretical calorie reductions, FoP traffic light colour and nutritional claims for different formulations.

Formulation	Theoretical Energy kcal/100 g	Theoretical Reduction of Calories %	Fibre Content Per Portion (baked ~75 g)	Sugar Content g/100 g and FoP Colour	Fat Content g/100 g and FoP Colour	Nutritional Claims European Commission [57]
Control	370	-	-	22.84	16.61	-
10_Sugar_Inulin	366	1.02%	1.87	20.55	16.61	N/A
30_Sugar_Inulin	358	3.06%	5.62	15.99	16.61	Reduced sugar/High in fibre ≥ 6 g/100 g
10_Fat_Inulin	359	3.03%	1.36	22.84	14.95	N/A
10_Fat_GBF	361	2.45%	1.36	22.84	14.95	N/A
30_Fat_Inulin	336	9.08%	4.10	22.84	11.63	Reduced fat/Source of fibre ≥ 3 g/100 g
30_Fat_GBF	342	7.36%	4.10	22.84	11.63	Reduced fat/Source of fibre ≥ 3 g/100 g
10_Sugar_Inulin_10_Fat_Inulin	355	4.05%	3.23	20.55	14.95	Source of fibre ≥ 3 g/100 g
10_Sugar_Inulin_10_Fat_GBF	357	3.47%	3.23	20.55	14.95	Source of fibre ≥ 3 g/100 g
30_Sugar_Inulin_30_Fat_Inulin	325	12.14%	9.70	15.99	11.63	Reduced sugar/Reduced fat/ High in fibre ≥ 6 g/100 g
30_Sugar_Inulin_30_Fat_GBF	331	10.42%	9.70	15.99	11.63	Reduced sugar/Reduced fat/ High in fibre ≥ 6 g/100 g

N/A: not applicable.

**Table 7:** Baseline characteristics of participants.

Parameter	All Participants (*n* = 13)
Sex (Male/Female)	12/1
Age (y)	31.4 ± 7
BMI (kg/m^2^)	24.7 ± 3.8
Height (m)	1.77 ± 0.08
Weight (kg)	78.3 ± 17.3
Fasting Blood Glucose (Mmol/L)	4.7 ± 0.2

**Table 8:** Microbial profile of control, 30_Sugar_Inulin and 30_Fat_Inulin (storage for 10 days at 25 °C).

**Yeasts and Moulds**			
Storage Day	Control (log CFU/g)	30_Sugar_Inulin (log CFU/g)	30_Fat_Inulin (log CFU/g)
1	<1	<1	<1
3	<1	<1	<1
7	<1	<1	<1
10	<1	<1	<1
Total Viable Count			
Storage day	Control (log CFU/g)	30_Sugar_Inulin (log CFU/g)	30_Fat_Inulin (log CFU/g)
1	<1	<1	<1
3	1.10 ± 0.17 ^a^	1.20 ± 0.35 ^a^	0.97 ± 0.85 ^a^
7	0.67 ± 0.58 ^a^	0.87 ± 0.81 ^a^	0.77 ± 0.68 ^a^
10	<1	<1	<1

Means within a row with the same letters are insignificant *p* < 0.05.

**Table 9:** The implication of using inulin and GBF in muffin formulations on retail cost.

Formulation	Fibre Content Per Portion (Baked ~75 g)	Additional Raw Material Cost Per Portion GBP £	Cost Per Retail Pack (4 Muffins) £
Control	-	-	1.18
10_Sugar_Inulin	1.87	0.041	1.34
30_Sugar_Inulin	5.62	0.124	1.67
10_Fat_Inulin	1.36	0.029	1.29
10_Fat_GBF	1.36	0.027	1.29
30_Fat_Inulin	4.10	0.086	1.52
30_Fat_GBF	4.10	0.082	1.51
10_Sugar_Inulin_10_Fat_Inulin	3.23	0.070	1.46
10_Sugar_Inulin_10_Fat_GBF	3.23	0.068	1.45
30_Sugar_Inulin_30_Fat_Inulin	9.70	0.210	2.02
30_Sugar_Inulin_30_Fat_GBF	9.70	0.206	2.00

## References

[1] Eknoyan G. (2006). A History of Obesity, or How What Was Good Became Ugly and Then Bad. Adv. Chronic Kidney Dis..

[2] Poti J.M., Braga B., Qin B. (2017). Ultra-processed Food Intake and Obesity: What Really Matters for Health—Processing or Nutrient Content?. Curr. Obes. Rep..

[3] Monteiro C.A., Moubarac J.-C., Levy R.B., Canella D., Louzada M.L.D.C., Cannon G. (2017). Household availability of ultra-processed foods and obesity in nineteen European countries. Public Heal. Nutr..

[4] Buttriss J.L. (2012). Food reformulation: The challenges to the food industry. Proc. Nutr. Soc..

[5] Norton I., Fryer P., Moore S. (2006). Product/Process integration in food manufacture: Engineering sustained health. AIChE J..

[6] Harastani R., James L.J., Walton J., Woolley E. (2020). Tackling obesity: A knowledge-base to enable industrial food reformulation. Innov. Food Sci. Emerg. Technol..

[7] Absolute Reports (2020). Global Muffins Market Research Report 2020. https://www.absolutereports.com/global-muffins-market-15040981.

[8] Irish Food Board [IFB] (2018). Healthy Snaking UK and Ireland. https://www.bordbia.ie/globalassets/bordbia.ie/industry/marketing-reports/consumer-reports/healthy-snacking-uk-and-ireland-january-2018.pdf.

[9] Hashem K.M., He F.J., Alderton S.A., MacGregor G.A. (2018). Cross-sectional survey of the amount of sugar and energy in cakes and biscuits on sale in the UK for the evaluation of the sugar-reduction programme. BMJ Open.

[10] Research and Markets (2017). Global Muffins Market 2017–2021. https://www.researchandmarkets.com/reports/4376211/global-muffins-market-2017-2021.

[11] World Health Organisation [WHO] (2018). Obesity and Overweight. https://www.who.int/news-room/fact-sheets/detail/obesity-and-overweight.

[12] Public Health England [PHE] (2017). Sugar Reduction: Achieving the 20%: A Technical Report Outlining Progress to Date, Guidelines for Industry, 2015 Baseline Levels in Key Foods and Next Steps. https://assets.publishing.service.gov.uk/government/uploads/system/uploads/attachment_data/file/604336/Sugar_reduction_achieving_the_20_.pdf.

[13] Asioli D., Aschemann-Witzel J., Caputo V., Vecchio R., Annunziata A., Næs T., Varela P. (2017). Making sense of the “clean label” trends: A review of consumer food choice behavior and discussion of industry implications. Food Res. Int..

[14] European Commission (2011). Regulation (EU) No 1131/2011: Amending Annex II to Regulation (EC) No 1333/2008 of the European Parliament and of the Council with Regard to Steviol Glycosides. Off. J. Eur. Union.

[15] European Commission (2018). Regulation (EU) 2018/97: Amending Annex II to Regulation (EC) No 1333/2008 of the European Parliament and of the Council as Regards the Use of Sweeteners in Fine Bakery Wares. Off. J. Eur. Union.

[16] Gao J., Brennan M., Mason S.L., Brennan C. (2016). Effect of sugar replacement with stevianna and inulin on the texture and predictive glycaemic response of muffins. Int. J. Food Sci. Technol..

[17] Miller R.A., Dann O.E., Oakley A.R., Angermayer M.E., Brackebusch K.H. (2017). Sucrose replacement in high ratio white layer cakes. J. Sci. Food Agric..

[18] Burgos K., Subramaniam P., Arthur J. (2016). Reformulation Guide Spotlight on Sugars for Small to Medium Sized Companies. Leatherhead Food Research. https://www.fdf.org.uk/corporate_pubs/Reformulation-Guide-Sugars-Aug2016.pdf.

[19] Struck S., Jaros D., Brennan C., Rohm H. (2014). Sugar replacement in sweetened bakery goods. Int. J. Food Sci. Technol..

[20] Brooker B.E. (1993). The Stabilisation of Air in Cake Batters—The Role of Fat. Food Struct.

[21] Zahn S., Pepke F., Rohm H. (2010). Effect of inulin as a fat replacer on texture and sensory properties of muffins. Int. J. Food Sci. Technol..

[22] Marchetti L., Califano A., Andrés S. (2018). Partial replacement of wheat flour by pecan nut expeller meal on bakery products. Effect on muffins quality. LWT.

[23] Oh I.K., Lee S. (2018). Utilization of foam structured hydroxypropyl methylcellulose for oleogels and their application as a solid fat replacer in muffins. Food Hydrocoll..

[24] Acosta K., Cavender G., Kerr W.L. (2011). Sensory and Physical properties of muffins made with waxy whole wheat flour. J. Food Qual..

[25] Sanz T., Salvador A., Baixauli R., Fiszman S.M. (2009). Evaluation of four types of resistant starch in muffins. II. Effects in texture, colour and consumer response. Eur. Food Res. Technol..

[26] Martínez-Cervera S., De La Hera E., Sanz T., Gomez M., Salvador A. (2011). Effect of using Erythritol as a Sucrose Replacer in Making Spanish Muffins Incorporating Xanthan Gum. Food Bioprocess Technol..

[27] Zahn S., Forker A., Krügel L., Rohm H. (2013). Combined use of rebaudioside A and fibres for partial sucrose replacement in muffins. LWT.

[28] Grigelmomiguel N., Carreras-Boladeras E., Martin-Belloso O. (2001). Influence of the Addition of Peach Dietary Fiber in Composition, Physical Properties and Acceptability of Reduced-Fat Muffins. Food Sci. Technol. Int..

[29] Duffrin M.W., Holben D.H., Bremner M.J. (2001). Consumer Acceptance of Pawpaw (*Asimina triloba*) Fruit Puree as a Fat-Reducing Agent in Muffins, Compared to Muffins Made with Applesauce and Fat. Fam. Consum. Sci. Res. J..

[30] Giacomozzi A.S., Carrín M.E., Palla C.A. (2018). Muffins Elaborated with Optimized Monoglycerides Oleogels: From Solid Fat Replacer Obtention to Product Quality Evaluation. J. Food Sci..

[31] Soong Y.Y., Quek R.Y.C., Henry C.J. (2015). Glycemic potency of muffins made with wheat, rice, corn, oat and barley flours: A comparative study between in vivo and in vitro. Eur. J. Nutr..

[32] Stewart M.L., Zimmer J.P. (2018). Postprandial glucose and insulin response to a high-fiber muffin top containing resistant starch type 4 in healthy adults: A double-blind, randomized, controlled trial. Nutrition.

[33] Rolls B.J., Drewnowski A., Ledikwe J. (2005). Changing the Energy Density of the Diet as a Strategy for Weight Management. J. Am. Diet. Assoc..

[34] Garcia J.R., Sahi S.S., Hernando I. (2014). Functionality of lipase and emulsifiers in low-fat cakes with inulin. LWT.

[35] Chetachukwu A.S., Thongraung C., Yupanqui C.T., Adegoke S.C. (2018). Effect of short-chain inulin on the rheological and sensory characteristics of reduced fat set coconut milk yoghurt. J. Texture Stud..

[36] Sarawong C., Schoenlechner R., Sekiguchi K., Berghofer E., Ng P.K. (2014). Effect of extrusion cooking on the physicochemical properties, resistant starch, phenolic content and antioxidant capacities of green banana flour. Food Chem..

[37] Aurore G., Parfait B., Fahrasmane L. (2009). Bananas, raw materials for making processed food products. Trends Food Sci. Technol..

[38] Alves L.A.A.D.S., Lorenzo J.M., Gonçalves C.A.A., dos Santos B.A., Heck R.T., Cichoski A.J., Campagnol P.C.B. (2016). Production of healthier bologna type sausages using pork skin and green banana flour as a fat replacers. Meat Sci..

[39] Kumar V., Biswas A.K., Sahoo J., Chatli M.K., Sivakumar S. (2011). Quality and storability of chicken nuggets formulated with green banana and soybean hulls flours. J. Food Sci. Technol..

[40] Wang Y., Zhang M., Mujumdar A.S. (2012). Influence of green banana flour substitution for cassava starch on the nutrition, color, texture and sensory quality in two types of snacks. LWT.

[41] Khoozani A.A., Kebede B., Bekhit A.E.-D.A. (2020). Rheological, textural and structural changes in dough and bread partially substituted with whole green banana flour. LWT.

[42] Karp S., Wyrwisz J., Kurek M., Wierzbicka A. (2016). Physical properties of muffins sweetened with steviol glycosides as the sucrose replacement. Food Sci. Biotechnol..

[43] Struck S., Gundel L., Zahn S., Rohm H. (2016). Fiber enriched reduced sugar muffins made from iso-viscous batters. LWT.

[44] Arifin N., Siti Nur Izyan M.A., Faujan N.H. (2019). Physical properties and consumer acceptability of basic muffin made from pumpkin puree as butter replacer. Food Res..

[45] Atwater W.O., Bryant A.P. (1899). The Availability and Fuel Values of Food Materials. Connecticut (Storrs) Agricultural Experiment Station 12th Annual Report.

[46] Food and Agriculture Organisation [FAO] (2003). Food Energy—Methods of Analysis and Conversion Factors. Report of a Technical Workshop, Rome. http://www.fao.org/3/Y5022E/Y5022E00.htm.

[47] Public Health England [PHE] (2019). Composition of Foods Integrated Dataset (CoFID). https://www.gov.uk/government/publications/composition-of-foods-integrated-dataset-cofid.

[48] USDA. U.S. Department of Agriculture Food Data Central. Food Composition Table. https://fdc.nal.usda.gov/.

[49] Brouns F., Bjorck I., Frayn K.N., Gibbs A.L., Lang V., Slama G., Wolever T.M.S. (2005). Glycaemic index methodology. Nutr. Res. Rev..

[50] Quílez J., Bulló M., Salas-Salvadó J. (2007). Improved Postprandial Response and Feeling of Satiety after Consumption of Low-Calorie Muffins with Maltitol and High-Amylose Corn Starch. J. Food Sci..

[51] Martínez-Cervera S., Salvador A., Sanz T. (2015). Cellulose ether emulsions as fat replacers in muffins: Rheological, thermal and textural properties. LWT.

[52] Martínez-Cervera S., Sanz T., Salvador A., Fiszman S. (2011). Rheological, textural and sensorial properties of low-sucrose muffins reformulated with sucralose/polydextrose. LWT.

[53] Pateras I.M.C., Rosenthal A. (1992). Effects of sucrose replacement by poly dextrose on the mechanism of structure formation in high ratio cakes. Int. J. Food Sci. Nutr..

[54] Sikorski Z.E., Sikorska-Wisniewska G., Williams C., Buttriss J. (2006). The role of lipids in food quality. Improving the Fat Content of Foods.

[55] Heo Y., Kim M.-J., Lee J.-W., Moon B. (2019). Muffins enriched with dietary fiber from kimchi by-product: Baking properties, physical-chemical properties, and consumer acceptance. Food Sci. Nutr..

[56] Martínez-Cervera S., Salvador A., Muguerza B., Moulay L., Fiszman S. (2010). Cocoa fibre and its application as a fat replacer in chocolate muffins. LWT.

[57] European Commission (2012). Nutrition Claims, Annex of Regulation (EC) No 1924/2006 Amended by Regulation (EU) No 1047/2012. https://ec.europa.eu/food/safety/labelling_nutrition/claims/nutrition_claims_en.

[58] Food Standards Agency [FSA] (2016). Guide to Creating a Front of Pack (FoP) Nutrition Label for Pre-Packed Products Sold through Retail Outlets. https://www.food.gov.uk/sites/default/files/media/document/fop-guidance_0.pdf.

[59] Singh J., Dartois A., Kaur L. (2010). Starch digestibility in food matrix: A review. Trends Food Sci. Technol..

[60] Guillon F., Champ M. (2000). Structural and physical properties of dietary fibres, and consequences of processing on human physiology. Food Res. Int..

[61] Brennan C.S., Kuri V., Tudorica C.M. (2004). Inulin-enriched pasta: Effects on textural properties and starch degradation. Food Chem..

[62] Borczak B., Sikora E., Sikora M., Rosell C.M., Collar C. (2012). Glycaemic response to frozen stored wheat rolls enriched with inulin and oat fibre. J. Cereal Sci..

[63] Lightowler H., Thondre S., Holz A., Theis S. (2017). Replacement of glycaemic carbohydrates by inulin-type fructans from chicory (oligofructose, inulin) reduces the postprandial blood glucose and insulin response to foods: Report of two double-blind, randomized, controlled trials. Eur. J. Nutr..

[64] Slavin J., Green H. (2007). Dietary fibre and satiety. Nutr. Bull..

[65] Archer B.J., Johnson S.K., Devereux H.M., Baxter A.L. (2004). Effect of fat replacement by inulin or lupin-kernel fibre on sausage patty acceptability, post-meal perceptions of satiety and food intake in men. Br. J. Nutr..

[66] Hiel S., Bindels L.B., Pachikian B.D., Kalala G., Broers V., Zamariola G., Chang B.P.I., Kambashi B., Rodriguez J., Cani P.D. (2019). Effects of a diet based on inulin-rich vegetables on gut health and nutritional behavior in healthy humans. Am. J. Clin. Nutr..

[67] Salmean Y.A. (2017). Acute fiber supplementation with inulin-type fructans curbs appetite sensations: A randomized, double-blind, placebo-controlled study. Food Nutr. Res..

[68] Smith J.P., Daifas D.P., El-Khoury W., Koukoutsis J., El-Khoury A. (2004). Shelf Life and Safety Concerns of Bakery Products—A Review. Crit. Rev. Food Sci. Nutr..

[69] Rodriguez M.V., Medina L.M., Jordano R. (2002). Prolongation of shelf life of sponge cakes using modified atmosphere packaging. Acta Aliment..

[70] Bryan F.L., Guzewich J.J., Todd E.C.D. (1997). Surveillance of Foodborne Disease III. Summary and Presentation of Data on Vehicles and Contributory Factors; Their Value and Limitations. J. Food Prot..

[71] Doulia D., Katsinis G., Rigas F. (2006). Prediction of the Mould-Free Shelf Life of Muffins. Int. J. Food Prop..

[72] Lund B. (1990). Foodborne disease due to Bacillus and Clostridium species. Lancet.

[73] Grabitske H.A., Slavin J.L. (2009). Gastrointestinal Effects of Low-Digestible Carbohydrates. Crit. Rev. Food Sci. Nutr..

[74] Bruhwyler J., Carreer F., Demanet E., Jacobs H. (2009). Digestive tolerance of inulin-type fructans: A double-blind, placebo-controlled, cross-over, dose-ranging, randomized study in healthy volunteers. Int. J. Food Sci. Nutr..

[75] Bonnema A.L., Kolberg L.W., Thomas W., Slavin J.L. (2010). Gastrointestinal Tolerance of Chicory Inulin Products. J. Am. Diet. Assoc..

[76] Marotta G., Simeone M., Nazzaro C. (2014). Product reformulation in the food system to improve food safety. Evaluation of policy interventions. Appetite.

